# Detection of squirrel poxvirus by nested and real-time PCR from red (*Sciurus vulgaris*) and grey (*Sciurus carolinensis*) squirrels

**DOI:** 10.1186/1746-6148-6-33

**Published:** 2010-06-08

**Authors:** Janus W Atkin, Alan D Radford, Karen P Coyne, Jenny Stavisky, Julian Chantrey

**Affiliations:** 1 Department of Veterinary Pathology, University of Liverpool, Leahurst Campus, Neston, Cheshire, CH64 7TE, UK; 2 Department of Veterinary Clinical Sciences, University of Liverpool, Leahurst Campus, Neston, Cheshire, CH64 7TE, UK

## Abstract

**Background:**

Squirrel poxvirus (SQPV) is highly pathogenic to red squirrels (*Sciurus vulgaris*), and is a significant contributing factor to the local extinction of the species in most parts of England and Wales, where infection is endemic in Eastern grey squirrel (*Sciurus carolinensis*) populations. Although a nested PCR assay has been used successfully to study the epidemiology of SQPV, samples have a long processing time and the assay is not quantifiable.

**Results:**

This project describes the design and optimization of a real-time PCR for SQPV. Comparison with the nested PCR showed the real-time assay to be more sensitive by one log and able to detect approximately 144 genome copies per mg of tissue.

**Conclusions:**

The real-time PCR has been used to quantify viral genome load in tissues from diseased and apparently healthy red and grey squirrels, and suggests that the titre of virus in tissues from diseased red squirrels is considerably higher than that found even in a grey squirrel with cutaneous lesions.

## Background

Squirrelpox is a disease caused by a virus of the family *Poxviridae*. Although originally described as a parapoxvirus, it is now considered to belong to a previously undescribed genus of the Chordopoxvirinae subfamily [1,2]. In the UK, squirrelpox virus (SQPV) is known to infect two species of squirrel: the native red squirrel (*Sciurus vulgaris*) and the introduced Eastern grey squirrel (*Sciurus carolinensis*) [3]. In red squirrels, the disease is characterized by a severe multifocal ulcerative and exudative dermatitis with hemorrhagic scabs which is predominantly fatal if untreated. Such lesions appear primarily on the eyelids, nose and lips, with later spread to the digits, inguinal area and ventral skin of the body [4-7]. Affected animals develop widespread epidermal necrosis, resulting in dehydration, a loss of body condition and ultimately death, although in experimental controlled conditions, one of four red squirrels were able to recover [8]. In contrast to red squirrels, grey squirrels generally do not show any clinical signs of infection [8]. Only one exceptional case of disease has been described in a grey squirrel with proliferative oral lesions from which poxviruses were demonstrated by electron microscopy [9].

The first recorded epidemics of pox-like disease in red squirrels occurred in the 1900 s [10] and continue to the present day [4,7,11]. The origins of SQPV are poorly understood, but it is suggested that the virus was introduced to Britain along with some groups of grey squirrels from North America, as outbreaks of squirrelpox were not reported until after their introduction [6]. In support of this theory a large proportion of grey squirrels are seropositive in different studies; 100% (7 of 7) of grey squirrels from Wisconsin USA [2], and 61% (136 of 223) of grey squirrels in the UK [3] were seropositive to SQPV suggesting that this species could be the reservoir host. In contrast, all other woodland rodent species tested in Britain (wood mice, *Apodemus sylvaticus*, and bank voles, *Clethrionomys glareolus*) were serologically negative [2].

Squirrelpox virus is now considered by many to be the main driving force behind the decline of the red squirrel in the UK, although competition with grey squirrels is also a factor [12]. Red squirrel populations have been shown to decline 17-25 times faster where they co-exist with grey squirrels with serological evidence of exposure to SQPV compared to where the grey squirrels are free of infection [13]. Currently, squirrelpox is advancing north into Scotland with grey squirrels moving from areas with endemic poxvirus [10] and therefore, there is an urgent need to develop tools to assist in understanding the epidemiology and pathogenesis of this poxvirus.

Although the presence of gross or histological lesions can help form a putative diagnosis of SQPV infections in red squirrels, other diagnostic tools are needed to confirm this, especially in early cases or those necropsy cases which are autolysed. Electron microscopy has been used previously to confirm infection in red squirrels [6,11,14] but is of limited use in grey squirrels where lesions are generally absent [3] and correspondingly virus production may be minimal. To diagnose squirrelpox in these animals, serological or molecular methods must be used. Despite ELISA being a useful diagnostic tool, it has limitations: assays can suffer from poor specificity or sensitivity, they identify past exposure rather than present infection, and they cannot detect the presence of SQPV in the environment, or in vectors. Assays based on the polymerase chain reaction (PCR) can overcome most of these problems and we have been using a nested PCR to monitor the epidemiology of squirrelpox in both red and grey squirrel populations (not published). This work aims to compare the sensitivity of this previously used nested PCR with a new real-time PCR and describes how the distribution and level of virus load in the tissues tested can be used to elucidate pathogenesis and potential transmission routes.

## Results

### Gross and histopathological findings

All four red squirrels, with gross lesions, showed multifocal moderate to severe chronic periorbital, nasal, perioral or buccal ulcerative and exudative dermatitis. Often submandibular lymph nodes were enlarged or reddened. Red squirrels showed less severe but similar multifocal cutaneous ulcerative lesions on the digits of the forepaws and other cutaneous sites. Histologically, skin lesions showed widespread severe ballooning degeneration of keratinocytes of the epidermis and hair follicles with multifocal ulceration, a serocellular crust and a perivascular dermal infiltrate of neutrophils and lymphocytes. Two cases (red squirrels 1 and 3) showed eosinophilic intracytoplasmic inclusion bodies in degenerating keratinocytes. Lesions were similar to those from previously described red squirrelpox cases [4,6,7]

Grey squirrel 1, with a gross buccal lesion, showed a focal mild chronic ulcerative cheilitis with a neutrophil rich serocellular crust and superficial bacteria. Red squirrel 5 and grey squirrels 2-4, which all lacked cutaneous gross lesions, were unremarkable histologically.

### Nested PCR results

For the five nested amplicons from three red and two grey squirrels that were sequenced, all were 100% identical to the published target sequence for the SQPV RNA polymerase subunit RPO147 gene (AY340975) (data not presented).

### Real-time PCR primer development

**Figure 1:**
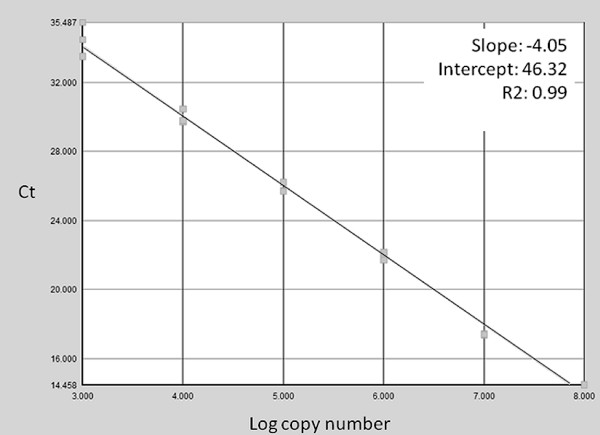
**Efficiency plot for the real-time PCR assay generated using the manufactured template control**. Log copy number is plotted against cycle threshold (Ct) value.

**Figure 2:**
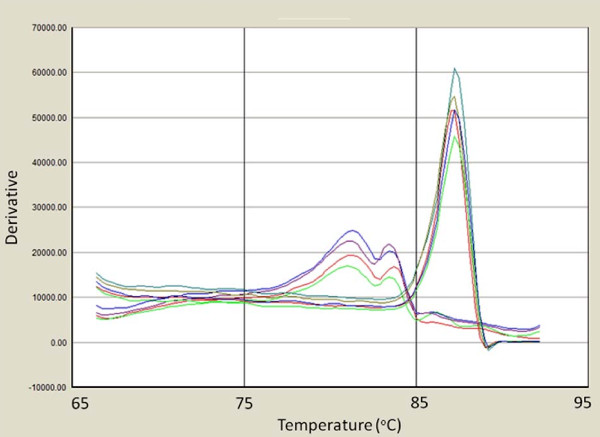
**Melt curve analysis (derivative - rate of change of fluorescence against temperature) for positive and negative amplicons from the real-time assay**. Amplicons from squirrel poxvirus gave a specific melt at approximately 88°C. Negative control samples gave distinguishably lower melt curves and are seen below between 76°C and 85°C.

Examples of the typical results of the real-time PCR efficiency plot and melt curve analysis are shown in figures 1 and 2 respectively. Taken as a whole, using the manufactured molecule template, the assay had a final sensitivity of approximately 36 synthetic genome copies per reaction, which equates to 144 genome copies per mg of tissue. SQPV amplicons gave a specific melt temperature of 87-88°C. Negative samples did occasionally show a product with a lower melting temperature suggestive of primer dimers. However, this was generally not apparent in samples that also tested positive for SQPV, and so was unlikely to significantly affect the estimated titre. The only other poxvirus to give a melt curve was orf virus, however this was at a clearly lower temperature from SQPV. The other three poxviruses failed to amplify, (myxoma, cowpox and bovine papular stomatitis viruses) all giving non-specific signals equivalent to negative controls (data not presented). These data suggest that although sensitive, the assay could benefit from further optimisation to reduce primer-dimer formation in negative controls and to increase efficiency.

### Comparison of real-time and nested PCR sensitivity

Using the 10-fold dilution series of DNA from red-squirrel tissue with lesions, the nested PCR was able to detect SQPV DNA serially diluted by 5 logs. In comparison, the real-time PCR showed a positive result at dilutions of 6 logs, suggesting that the real-time PCR is approximately 10-times more sensitive than the nested assay.

The ability of the two assays to detect SQPV DNA in grey squirrel tissues expected to have low SQPV titres are summarised in table 1. When the equivocal real-time PCR results are ignored (4), the agreement between the results from the two assays is 80% (28/35). However, for the discordant results, 9% (3/35) of the samples tested real-time positive/nested PCR negative whereas 11% (4/35) of the samples tested real-time negative/nested PCR positive.

**Table 1:** Comparison of nested and real-time PCR results from 39 grey skin samples

		Nested PCR results	
		
		Positive	Negative
Real-time PCR results	Positive^a^	**13**	3
	
	Negative^b^	4	**15**
	
	Equivocal^c^	1	3

^a ^3/3 or 2/3 of the triplicates positive Ct and appropriate melt curve.

^b ^0/3 positive Ct and no melt curve.

^c ^1/3 positive Ct and melt curve.

### Virus distribution and quantification

**Figure 3:**
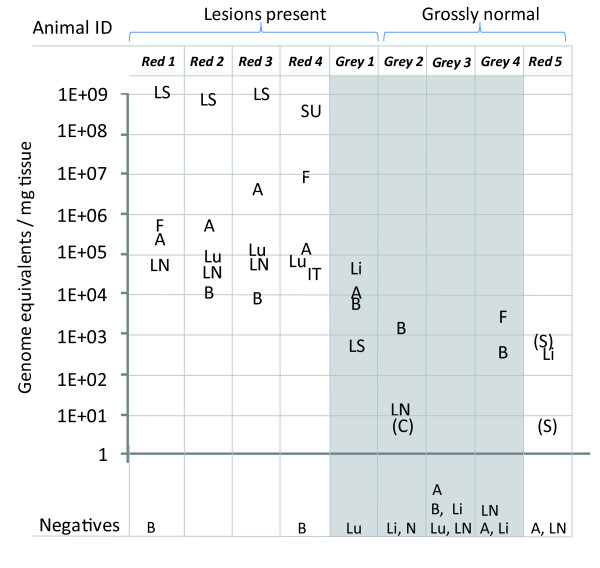
**Real-time PCR results showing titre in copies/mg from tissue or flea samples of five red and four grey squirrels**. Squirrel tissues tested were as follows: A (antebrachial skin), B (blood), C (cheek), IT (intestinal tract), Li (lip), LS (lip scab or ulcer), LN (submandibular lymph node), Lu (lung), N (nasal skin), SU (eyelid skin ulcer), S (facial skin) and additionally fleas (F) when present. Tissues are without gross lesions unless indicated. Positive samples are indicated in the figure, and are expressed as the average of three reactions. Those samples still testing equivocal on repeat are indicated by brackets. Samples testing negative are shown below the figure.

The SQPV tissue distribution in the necropsy samples from five red and four grey squirrels (with and without gross squirrelpox lesions) is shown in figure 3. Almost all tissues from red squirrels with SQPV lesions were found to be unequivocally positive by the real-time PCR, with all tissues testing positive showing relatively high titres. The highest viral DNA concentrations were generally found in the lip, with lower viral concentrations found in other tissues, such as submandibular lymph node, lung and blood. The average DNA load in the positive tissues from the grossly affected red squirrels was very high (2 × 10^8 ^copies/mg tissue (figure 3). The average DNA concentration in the positive tissues from the grey squirrel with lesions (grey 1) was 1 × 10^4 ^lower than in the red squirrels with lesions. The average DNA load in the positive tissues from grossly normal animals (grey squirrels 2, 3, 4 and red squirrel 5) was 2 × 10^6 ^lower than in the red squirrels with lesions. All three groups of fleas from two red (red squirrels 1 and 4) and one grey squirrel (grey squirrel 4) showed evidence of viral DNA at comparatively high concentrations for those squirrel species.

## Discussion

Squirrel poxvirus is one of the major threats to the red squirrel in the UK [14]. Here we describe a nested and real-time PCR assay, both of which were able to detect poxviral DNA from tissues from red and grey squirrels naturally infected with squirrel pox virus. The real-time PCR could identify SQPV DNA in a wider range of tissues as compared to the nested PCR, including blood from grey squirrels. It could also detect lower concentrations of DNA from the diluted red squirrel tissue with lesions making it a more sensitive diagnostic test. Real-time PCR quantification suggests detection is sensitive to approximately 144 genome equivalents per mg tissue which is comparable with other real-time poxviral assays [15].

Overall, the concordance between real-time PCR and nested PCR assays results, for grey skin samples, was approximately 80%, excluding the four borderline real-time PCR cases that still produced equivocal results despite retesting. Probably these cases represent those with very low viral DNA loads but this was a more stringent comparison than conducting the test using red squirrel tissue with lesions. Given there is currently no definitive way to identify viral infection in grey squirrels (no gold standard), it is not possible to know which test gives the more accurate indication of the true infection status. The three samples which tested real-time PCR positive and nested PCR negative are likely due to increased test sensitivity using the real time assay. The four samples which tested real-time PCR negative and nested PCR positive could be due to either degradation of DNA samples from successive free-thaw cycles or possibly contamination, a known problem of such nested assays, and one of the main drivers for us to develop the real-time assay. Periodically during testing, nested PCR negative controls occasionally would show positive results, in which case, the entire experiment was repeated. However, contamination between individual test samples is a possible but unlikely scenario. Selective inhibition of the real-time PCR compared to the nested PCR is similarly improbable. An endogenous amplification control was not included in the assay due to the diverse nature of tissue samples being tested, since any internal control based on the squirrel genome would have to be individually optimised for each tissue type. Therefore our interpretation of the data is based upon a pattern of infection across several animals and several tissues rather than relying on any one individual result. Correspondingly, false negative results are a possibility.

The results from the four red squirrels with lesions suggest that SQPV undergoes systemic spread in this species, with a large majority of samples testing positive and viral DNA being found in all tissue types tested. Systemic spread is a common feature of some poxviruses including ectromelia in mice [16], cowpox virus in certain felines[17,18], and myxoma virus in the European rabbit (*Oryctolagus cuniculus*)[19]. All these poxviruses are benign or show limited infections in their natural host yet produce severe disease in a novel host. In the red squirrel, poxviral damage to the skin produces ballooning degeneration of keratinocytes, epithelial necrosis and then central ulceration [6,7]. Therefore, these cutaneous sites are highly productive sites for viral replication. Unsurprisingly, these tissues with the gross and histological lesions have the most abundant viral DNA using this real-time PCR. As with ectromelia, viraemia probably allows the virus to disperse to multifocal skin sites where the most productive replication takes place and transmission occurs [16]. In contrast, tissues without gross lesions, for example lung, contain less viral DNA.

For red squirrels, the consistently high copy numbers in ulcerated lip and skin samples support evidence from nested PCR studies, gross and histopathology, that tissue from these sites are the most reliable for use in squirrelpox diagnosis in red squirrels[6,7,14]. Lesser amounts of viral DNA are present in the grossly normal skin (antebrachium), submandibular lymph nodes and other organs. Blood was variably positive and at a lower concentration compared with other red squirrel tissues. This may reflect the stage of infection with relatively low levels of viraemia coinciding with peak clinical disease [20]. However, other factors such as PCR inhibitors in blood cannot be excluded[21].

Finding grey squirrels with SQPV induced lesions appears extremely rare. Indeed, in the authors' experience, only one such animal has been identified out of 138 screened at necropsy (data not presented). Qualitatively, SQPV-infection in samples from this grey squirrel with lesions appeared similar to those for positive red squirrels, with five out of six samples testing positive. However, quantitatively, the amount of viral DNA detected in this grey squirrel was on average 1 × 10^4 ^lower than in the red squirrels with lesions, with blood and then lip having the highest titres (compared to lip samples from SQPV positive red squirrels). This suggests that even in the rare cases where SQPV is associated with disease in grey squirrels, viral replication is limited by as yet unknown mechanisms. The real-time PCR results from samples taken from grossly normal grey squirrel 3 strongly suggest that this animal is negative for SQPV. However, results from grey squirrels 2 (also positive by nested PCR for SQPV) and 4, (previously not tested by nested PCR) suggest that these are both weakly positive for SQPV as two samples were consistently positive for each animal on repeated testing. The precise significance of these results needs further clarification. Possibilities include that these animals are undergoing current sub-clinical infection and so acting as asymptomatic reservoirs, or that these PCR results represent non-infectious genome remnants persisting beyond the presence of infectious virus particles.

In addition to amplifying SQPV viral DNA from grossly normal grey squirrels, SQPV was also amplified from the lip of a grossly normal red squirrel 5 (two other skin samples tested equivocally positive). This animal had been accidentally killed on the road and had no gross nor histological lesions indicative of SQPV infection, and may represent a very early case of infection which was killed before significant lesions could develop. Alternatively, though less likely, it could be that this is a recovering red squirrel harbouring a limited degree of viral DNA during the healing process. In experimental SQPV infection of four red squirrels, one animal recovered from the disease [8] and seropositive red squirrel carcases have been occasionally recovered from the wild [14] so there is a remote possibility of a small number of immune individuals surviving however the overwhelming epidemiological picture is of rapid local and regional extinction of the red squirrel population [4,11]. Clearly, these low-titre positive samples from grossly normal animals are very close to the limit of detection for this assay and may need further validation in the context of larger sample numbers. There is the possibility that there may be less virulent squirrel poxvirus strains circulating in the population though there is very limited epidemiological evidence to support this [14].

An interesting point to note is that all three group flea samples tested (from red squirrels 1 and 4 with lesions, grossly normal grey squirrel 4) had relatively high concentrations of viral DNA. This could suggest that the flea may have the capability to act as a vector of squirrel poxvirus, as has already been shown for the related myxoma virus in rabbits [22,23]. In addition, swine poxvirus can be spread by invertebrate vectors [24] which reinforces this possibility of a flea poxvirus vector. Further work is needed to characterise the precise relationship between the squirrel flea and SQPV, and the role of the flea in the epidemiology of this disease. Clearly, a flea could test PCR positive as a result of viral mechanical contamination of the flea's ectoskeleton. However, it could also be as a result of ingesting a blood meal containing virus (transport host), or by the virus replicating in the ectoparasite's tissue (intermediate host). Unfortunately, many fleas had abandoned the carcasses before body recovery was possible so data was incomplete and more testing is necessary. In the UK, both the red squirrel flea (*Monopsyllus sciurorum sciurorum*) and the grey squirrel flea (*Orchopeas howardi howardi*) can be found on either species of squirrel [25,26].

In addition to a possible role for fleas in transmission, the presence of widespread viral DNA in many tissues from red squirrels suggests several potential routes of transmission are possible including superficial contamination of feeding sites, territorial marking sites, aerosols, faecal-oral routes and direct contact. Red squirrels with skin ulcers appear to be strongly positive for poxviral DNA and any contact with these ulcerated cutaneous sites could potentially lead to virus transmission.

In grey squirrels, the lip and antebrachial skin were primarily the main positive tissues in the grey squirrel with lesions and other cutaneous sites were largely negative (data not shown) whereas blood would appear to be the most consistent tissue to use by this method for detection of SQPV in grossly normal grey squirrels. In grey squirrels, viraemia occurred at relatively low concentrations and viral DNA was also present in lymph nodes and fleas which indicated systemic involvement although no clinical disease nor gross pathological lesions were apparent. Methods of viral spread in this species may be primarily ectoparasite driven or require a brief period of localised cutaneous infection with limited or no gross lesions. The duration of infection remains open to speculation but to have this relatively high percentage of grey squirrels testing positive suggests low level states of prolonged infection [27] or possibly sustained exposure to infection in the same individual, especially during periods of epidemic disease in red squirrels. This study also suggest that current grey squirrel culling regimes should consider sampling lip, antebrachial skin, blood and fleas to screen for SQPV infection status. When screening live grey squirrels, oral swabs may be sufficient but issues with lower sensitivity potentially could be problematic even by nested PCR.

A selection of amplicons from both assays were sequenced and shown to be identical for both red and grey squirrels although, occasional low concentration amplicons from tissues of both species failed to produce a product which could be sequenced. Although each amplicon was short and poxviruses tend to be genetically conserved in these regions [28], this further supports the hypothesis that this is the same poxvirus circulating between red and grey squirrels.

## Conclusions

We have designed a sensitive and specific real-time assay that is capable of giving quantitative data in SQPV infections. This assay will allow us to better understand the epidemiology and pathogenesis of this important disease. The main difference between SQPV positive red squirrel tissues and positive grey squirrel tissue is that grey squirrel samples contain considerably less viral DNA than the corresponding red squirrel samples (by several orders of magnitude). Another major difference is that, in red squirrels with lesions, the majority of tissue samples (and fleas) analysed will be positive, whereas even in a positive grey squirrel, most tissue samples are likely to be negative, with only a few sites being positive. There are, however, some similarities between the virus distribution in red and grey squirrels. From the results is seems that in both species, SQPV can infect the oral skin and lip and from there possibly spread to the submandibular lymph nodes and into the blood, and fleas from both species can carry the virus (possibly acting as vectors). The real time assay described here will allow us to further characterise the pathogenesis and epidemiology of this important disease.

## Methods

### Source material

During 2008, red squirrel bodies were recovered from the Formby National Trust reserve (UK grid reference SD281082) and grey squirrels were culled from controlled sites within a 10 km radius of the reserve. Grey squirrels culls occur routinely in the UK in areas with mixed squirrel populations, hence there was no need for a licence nor special ethical approval. Both carcases from red squirrels which had died naturally and culled grey squirrels were freely donated for scientific investigation by the Formby National Trust reserve. Bodies were either submitted fresh (having been refrigerated at 4°C until necropsy) or frozen at -20°C on collection and stored frozen until defrosting before post mortem examinations were performed. Given the relatively low number of squirrels involved in this study, it was possible to select those individuals with minimal levels of autolysis. At necropsy, all major organs including haemolysed blood were examined and sampled with tissues including multiple cutaneous sites such as the antebrachium and the lip. Tissue sections in 10% formal saline were prepared, embedded in paraffin wax for histopathological examination and stained with haematoxylin and eosin (H+E).

Due to prior experience with the nested PCR (unpublished data) showing only a very limited number of grey squirrel positive tissues having any evidence of viral DNA (exclusively skin sites and no other organs) and given the predominance of cutaneous lesions in red squirrels, skin and submandibular lymph nodes were preferentially selected for real-time PCR investigation. In addition, because of the potential for transmission of the virus by ectoparasites, as is the case for myxoma virus [29], prior to post-mortem examination, bodies were routinely combed and all fleas identified were harvested (up to a maximum of 10). Fleas were only available for three of the squirrels sampled and no other ectoparasite species were identified. Occasionally other internal tissues were sampled such as lung or intestinal tract when fleas, blood or lymph nodes were absent.

### Extraction of DNA

All DNA templates were extracted from 25 mg of squirrel tissue samples, or 100 ul of blood, or from crushed whole flea bodies, following the manufacturer's standard protocol (QIAGEN DNeasy Blood and Tissue Kit). During the final stage of preparation, the extracted DNA was eluted into 100 μL of elution buffer to increase final DNA concentration (as recommended by the standard protocol). DNA-extraction, negative-controls were included in all experiments (approximately one negative control per five samples). In the rare cases where negative controls tested positive, the entire experiment was repeated.

### Nested PCR

The primers used in the nested PCR target a section of a SQPV gene encoding the highly conserved RNA polymerase subunit RPO147 (accession number AY340975) (Table 2) [28]. The reaction conditions used in both rounds of the nested PCR consisted of the following: 20.5 μL 2× PCR Master Mix (1.5 mM MgCl_2_) (Thermo Scientific), 900 nM of each primer and 1.5 μL DNA template, giving a total of 25 μL per sample. The product from the 1^st ^round was diluted 1:20 with molecular grade water and the resulting mixture used as the 2^nd ^round DNA template.

**Table 2:** Primers used in the nested and real-time squirrelpox virus PCRs.

Stage	Primer orientation	Sequence (5' - 3')
1^st ^round nested PCR	SqP1 - forward SqP2 - reverse	GCGGCCGCGCTGACCGCCATCG CCAGCACGACTTCTTCCTGGAG

2^nd ^round nested PCR	SqP3 - forward SqP4 - reverse	CGCTCGCGTGTCCTACAGCCTG GCAGTCCGCAGCGCGCGCAGAT

Real-time PCR primers	SP7 - forward SP8 - reverse	GGGCGATCGTGCCGCTCAG CGCCTCGAGGTCCGGCTC

Both rounds of the PCR were carried out under the same cycling conditions: an initial denaturation stage at 95°C for 2 minutes; 35 cycles consisting of a denaturation stage at 95°C for 20 seconds, an annealing stage at 65°C for 20 seconds and an extension stage at 72°C for 1 minute; and a final extension stage at 72°C for 5 minutes. The products were electrophoretically resolved on 1% (w/v) agarose gels, then visualised under UV light after staining with ethidium bromide. The final amplicon of 275 base pairs from red squirrels 1, 2, and 3, and grey squirrrels 1 and 2, was sequenced to confirm the assay specificity. Amplicons were purified (Qiagen, QIAquick PCR purification kit) and their concentration standardised (Labtech nanodrop ND1000), before sequencing in both directions with the PCR primers (Macrogen).

### Real-time PCR

The target for the real-time PCR was a putative late gene transcription factor homologous to G8R in Vaccinia virus. The sequence of the SQPV G8R homologue was identified as part of an on-going effort to sequence the full SQPV genome [30]. The G8R homologue was chosen as it is pox specific, whilst providing several potential primer binding sites that are relatively conserved within several pox viruses. This minimised the chances of primer mismatch within SQPV whilst allowing for melt-curve differentiation of other poxviruses. The SQPV gene was compared to the analogous Orf virus gene (AY386264), and only regions that were highly conserved between the two species were chosen as primer candidates. Oligocalc was used to check primer candidates for self-complementarity and to ensure that pairs of primers had similar melting temperatures (Oligo Calculator version 3.23).

To optimise the assay, four pairs of primers were evaluated in early experiments and the best pair (SP7 5'-GGGCGATCGTGCCGCTCAG-3'/SP8 5'-CGCCTCGAGGTCCGGCTC-3') selected based on observed cycle threshold (Ct) value for signal fluorescence to exceed those of background controls, using a SQPV-positive control sample. Subsequent optimisation of the SP7/8 assay consisted of an annealing temperature gradient (55°C-70°C), and a primer gradient (50 nM, 100 nM, 200 nM and 300 nM). The final optimised PCR consisted of: 12.5 μL Absolute QPCR SYBR Green Mix (2×), 100 nM final concentration of each primer, 10.5 μL molecular grade water, and 1 μL DNA template. Reactions were run on an Applied Biosystems 7300 real-time cycler, with the following cycling conditions: an initial enzyme activation at 95°C for 15 minutes; 40 cycles consisting of denature (95°C for 30 seconds), anneal (68°C for 30 seconds), and extension (72°C for 1 minute); and a final extension of 72°C for 5 minutes. A melt curve was performed on all samples immediately after the final extension stage using the standard AB7300 melt curve. All samples were run in triplicate, and one non-template negative control was included for each sample. Positive reactions were those that had both a detectable Ct value and a melt curve in the range 87 to 88 degrees C. The reproducibility and interpretation of this assay was determined by running a selection of samples that were variably positive i.e. 3 of 3 reactions positive, 2 of 3 reactions positive, 1 of 3 reactions positive and all reactions negative. Based on these repeats, assay samples were subsequently indicated as positive when all three samples tested positive on a single assay, and negative when all samples were negative on a single assay. Samples where one or two samples tested positive were repeated. These samples were called positive if all three of the repeats tested positive, negative if all three tested negative and equivocal if one or two tested positive. Only three of the 46 samples tested fell into this latter category; rather than reporting these as negative these values have been included for completeness and are indicated as equivocal.

To confirm assay specificity, the strongest signal amplicons from selected reactions were sequenced. Those were taken from the skin lesions of red squirrels 1, 2, 3 and grey squirrel 1 and blood from grey squirrels 2 and 4. In addition, poxvirus lesion samples from cases of myxomatosis, bovine popular stomatitis, orf and cowpox were also tested by the real-time assay. These viruses are those that are closest phylogenetically to SQPV [2], and were derived from lesion material from a European rabbit (*Oryctolagus cuniculus*) with a swollen eyelid (myxoma virus) and a cheetah (*Acinonyx jubatus*) with a cutaneous scab (cowpox virus). Other poxvirus source material came from oral scabs from a sheep (containing orf virus) and a cow (containing bovine papular stomatitis virus) (both from D. Everest, VLA Weybridge).

### Comparison of nested and real-time PCR assays

Two approaches with different templates were used to compare the sensitivity of these two assays. Firstly, using a 10-fold dilution series of DNA from a highly-positive red squirrel lesion prepared in PCR-negative grey squirrel DNA. Secondly, using 39 DNA samples from culled grey squirrels' skin that had previously been assayed by nested PCR as having either low-range positive or negative viral titres.

### Virus distribution and quantification

To investigate virus distribution, necropsies were performed on nine squirrel carcasses, with five or six samples taken from varying sites on each squirrel according to lesion distribution and tissue availability. The nine squirrels included four red squirrels with lesions, one grey squirrel with lesions, three grossly normal grey squirrels, and one grossly normal red squirrel. All were taken from the Formby National Trust reserve, an area known to have endemic SQPV disease in 2008. From each sample, DNA was extracted and run on the real-time PCR assay.

In order to allow the estimation of genomic equivalents in these samples, a 143base artificial target sequence identical to the SQPV genome was manufactured for the real time assay (Bioneer). (5'-GGGCGATCGTGCCGCTCAGCGCCAGCGTCTGCTTCGACGGCGACACCAACTGCGTGTTCAACCTCCCGGTGCTCAAGGTCAAGAACTG CCTCTGCAGCTTCCACAGCGACGCCATGATCTCCATCGAGCCGGACCTCGAGGCG-3'). Serial 10-fold dilutions of this molecule were initially prepared in both negative squirrel DNA and water. However, since these two approaches gave identical results (data not presented), only water dilutions were used for subsequent experiments. The artificial target sequence was included as a dilution series ranging from 10^8 ^- 10^4 ^molecules per assay on each real-time PCR plate run. Applied Biosystems 7300 System SDS Software was used to construct a standard curve which was used to determine the approximate number of molecules of the target sequence in each pathological squirrel samples. Where the C_t _value fell outside the standard curve, these relatively low titres were estimated by extrapolation, and therefore their reported titres are likely to be a less precise reflection of the actual concentration of viral DNA in the tissues. All results were adjusted for differences in initial sample weights, and displayed in estimated number of copies (genome equivalents) per mg of tissue.

## Authors' contributions

JWA carried out the real-time PCRs and molecular lab work, collated the results and helped to draft the manuscript. JS and KPC participated in supervison of JWA on a daily basis, with interpretation of the results. ADR helped conceive the study, supervised JWA, designed the primers and many of the experiments, and helped write the manuscript. JC designed and coordinated the study and wrote the manuscript. All authors read and approved the final manuscript.
